# Safety and effectiveness using dexmedetomidine versus propofol TCI sedation during oesophagus interventions: a randomized trial

**DOI:** 10.1186/1471-230X-13-176

**Published:** 2013-12-30

**Authors:** Susanne Eberl, Benedikt Preckel, Jacques J Bergman, Markus W Hollmann

**Affiliations:** 1 Department of Anesthesiology, Academic Medical Center, University of Amsterdam, Meibergdreef 9, Amsterdam 1100 DD, The Netherlands; 2 Department of Gastroenterology & Hepatology, Academic Medical Center, University of Amsterdam, Meibergdreef 9, Amsterdam 1100 DD, The Netherlands

**Keywords:** Procedural sedation, Dexmedetomidine, Endoscopic oesophageal intervention

## Abstract

**Background:**

Endoscopic treatment of early neoplastic lesions in oesophagus has evolved as a valid and less invasive alternative to surgical resection. These endoscopic interventions are minimal invasive treatment options usually done with sedation on an outpatient basis. The aim of this trial is to determine the safety and effectiveness of dexmedetomidine sedation compared to the standard used propofol TCI sedation during endoscopic oesophageal interventions.

**Methods:**

The study will be performed as a randomized controlled trial. The first 64 consenting patients will be randomized to either the propofol or the dexmedetomidine group. Following endoscopy patients and gastroenterologists have to fill in questionnaires (PSSI, CSSI) (see abbreviations) about their sedation experiences. Additionally, patients have to accomplish the Trieger test before and after the procedure. Patient monitoring includes time adapted HR, SO_2_, ECG, NIBP, exCO_2,_ NICO, sweat conductance measurement, OAA/S, and the Aldrete score. Effectiveness of sedation, classified by satisfaction levels and pain and sedation score measured by questionnaires is the primary outcome parameter. Respiratory and hemodynamic complications are surrogate parameters for the secondary outcome parameter “safety”.

**Discussion:**

The acceptance level among patients after propofol sedation is high. Dexmedetomidine is a relatively new representative for procedural sedation. Has this new form of conscious sedation the potential to be safer and more effective for patients and endoscopists than propofol during endoscopic oesophageal interventions?

**Trial registration:**

This trial is registered in the ISRCTN Register (ISRCTN 68599804). It will be conducted in accordance with the protocol and in compliance with the moral, ethical, and scientific principles governing clinical research as set out in the Declaration of Helsinki (1989) and Good Clinical Practice (GCP). The Departments of Anesthesiology and Gastroenterology & Hepatology of the Academic Medical Center of Amsterdam are responsible for the design and conduct of the trial.

## Background

Early neoplasias in Barrett’s oesophagus bear the risk of developing carcinoma (5.1–7 per 1000 person-years) [1-3]. The last two decades, endoscopic treatment has evolved as a valid and less invasive alternative to surgical resection in patients with a low risk of lymph node metastasis [4,5].

The cornerstone of endoscopic therapy is endoscopic resection (ER), allowing curative removal and histological staging of neoplasia. To prevent recurrences it is important to eradicate all intestinal metaplasia. One approach is radiofrequency ablation (RFA) [6].

However, these procedures are long lasting, uncomfortable and stressful for most patients. Furthermore, they require patients being sedated, but easily arousable in order to provide excellent view to the oesophagus. Conscious sedation is a strategy for improving patient safety and comfort during these procedures. In particular, propofol is known to provide excellent sedation with a rapid onset and termination of action [7]. However, the most important disadvantage of propofol is the risk of a rapid change from conscious to deep sedation or even general anesthesia with consecutive cardiopulmonary depression [8]. Therefore, other pharmacological agents that induce an adequate level of sedation without respiratory depression are of increasing interest to clinicians. Dexmedetomidine, a short-acting selective alpha_2_-agonist, possesses anxiolytic, hypnotic, and analgesic properties [9]. Patients receiving dexmedetomidine are easily arousable, yet appear calm and comfortable. When they remain unstimulated, patients return to a hypnotic state. Furthermore, dexmedetomidine provides hemodynamic stability and appears to have no clinically important adverse effects on respiration [10].

The aim of this trial is to determine the safety and effectiveness of dexmedetomidine compared to the in our hospital standard used propofol sedation, both administered by anesthesia nurses.

## Methods/Design

### Trial design

The study is designed as a prospective randomized controlled trial.

### Participants

The study takes place at the Department of Gastroenterology and Hepatology in the Academic Medical Center (AMC) of the University of Amsterdam beginning July 2012 to Augustus 2013. Eligible patients for participation in this clinical trial are those planned to undergo elective endoscopic oesophageal interventions (mapping, ER, or RFA), aged above 18 years, and ASA classification I-III, who give written informed consent.

### Exclusion criteria

• Age range < 18 years

• ASA classification IV and V

• Allergic reaction to planned medication in the patients’ medical history

• Unregulated hypertension

• Hypovolemia or hypotension (systolic blood pressure <80 or mean arterial pressure <50 mmHg)

• Severe bradycardia (heart rate < 50/min) and / or related brady-dysrhythmias (e.g. advanced heart block)

• Impaired ventricular function (left ventricular ejection fraction <30%)

• Impaired renal function, GFR less than 15ml/min or undergoing hemodialysis

• Impaired liver function

• Substance abuse

The number of excluded patients and the reasons for their exclusion will be recorded and also reported in the manuscript according to the CONSORT statement.

### Informed consent

Patients scheduled for an elective endoscopic oesophageal intervention (mapping, ER, or RFA) will be asked – after checking inclusion and exclusion criteria - by phone to participate. The exclusion criteria will be checked once again during this conversation and if they are eligible for inclusion and interested in participating in our study, the patient information will be sent. Patients are included after written informed consent. Patients are free to participate in this study. If they deny taking part they will get the standard sedation with propofol and alfentanil provided by an anesthesia nurse as usual in daily practice for endoscopic oesophageal interventions. Patients don’t have to answer questionnaires, however - reflecting common monitoring practice - SO_2_, ECG, NIBP and exCO_2_ will be monitored in the same way.

### Randomization

**Figure 1:**
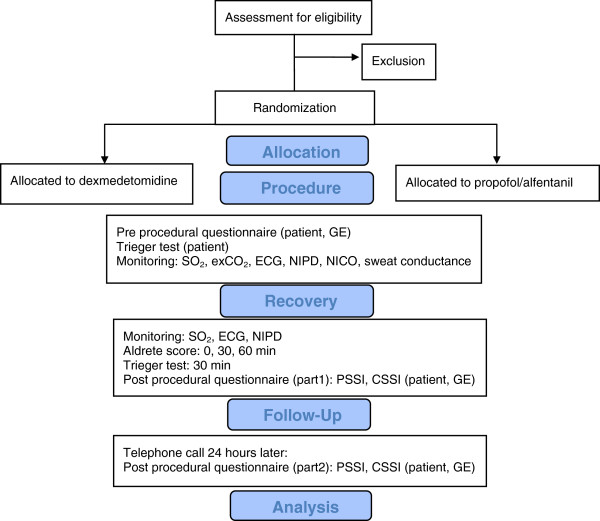
Consort flow diagram.

In the AMC, endoscopic oesophageal interventions are routine procedures performed on Monday and Friday on one intervention suite at the Department of Gastroenterology & Hepatology. Following informed consent, patients are computer generated randomized 1:1 to either: propofol TCI (group 1, n = 32) or dexmedetomidine (group 2, n = 32) (Figure 1).

Patients are blinded to the sedation regimen they are supposed to get.

Sedation within both groups is performed by a special anesthesia nurse who will not be blinded to the used form of sedation. The other attending parties (endoscopist, endoscopic nurse) are blinded.

All interventions are performed by one of two experienced endoscopists.

### Intervention

After randomization patients are allocated to group 1 or 2, and prepared for endoscopy. An intravenous line is inserted, and 500 ml NaCl 0,9%, glycopyrrolate 0.2 mg and lidocaine 50 mg are administered. Five minutes before insertion of the endoscope, the pharynx is sprayed with 10% xylocaine spray (xylocaine 10%, Astra). 2 L/min of oxygen will be administered by a nasal mask from start of sedation till the end of the endoscopic procedure.

Group 1 will receive sedation using a propofol Target Controlled Infusion (TCI) system. This TCI system is weight and age adapted pre-programmed using the Marsh pharmacokinetic model to attain a user defined propofol blood target level.

Sedation among group 2 will be addressed with dexmedetomidine.

Dexmedetomidine (Dexdor®: Orion corporation, Finland) will be started with a loading dose of 1 μg/kg intravenously over 10 min. After this loading bolus, procedure is started and dexmedetomidine continued throughout the procedure within the range of 0.7 -1 μg/kg/h titrated to a targeted level of sedation [10]. Among patients older than 65 years loading dose will be reduced to 0.5 μg/kg. continued throughout the procedure within the range of 0.7 -1 μg/kg/h titrated to a targeted level of sedation [10]. Among patients older than 65 years loading dose will be reduced to 0.5 μg/kg.

Both forms of sedation are supplied by an anaesthesia nurse to achieve the targeted sedation score (Observer’s Assessment of Alertness/Sedation OAAS Scale ≤ 4), which means the patients maximal lethargic response to their name spoken in normal tone.

If OAA/S > 4, which means that the patient is too alert or agitated to tolerate the procedure, additional sedation will be provided with step up of the TCI (group 1) or an additional doses of propofol 20 mg (group 2).

Drug infusion will be discontinued if any of the following adverse events are observed: recurrent apnea (respiratory rate <6/min) lasting more than 60 seconds over a 5 min observation period, sustained episodes (60 seconds) SO_2_ < 90% over a 5 min observation period, decrease of heart rate to < 50 beats/min, mean non-invasive blood pressure < 70% of the initial measurement.

### Monitoring

Reflecting common practice, patients are constantly monitored during endoscopy with HR, SO_2_, ECG, NIBP, and exCO_2_ measured at 5-minute intervals. Additionally sweat production as a sign of stress will be recorded with a wearable ambulatory skin conductance measurement and recording tool suitable for monitoring hot flashes. Furthermore, non invasive cardiac output (NICO) is measured using a Nexfin monitor.

We will record total procedure time, all drugs, drug amounts and time of administration, time from the end of the procedure until Observer’s Assessment of Alertness/Sedation (OAA/S) score ≥ 4, any respiratory and cardiovascular problems and all actions visibly taken to prevent or treat these problems, such as chin lift/jaw thrust, stimulating the patient, temporary mask ventilation, etc. The following were considered as significant events: a decline in SpO_2_ to less than 90%, breathing frequency less than 6 breath/min, or an increase of etCO_2_ > 50 mmHg; a change in heart rate to less or more than 20% of baseline or occurrence of any arrhythmia’s; blood pressure less or more than 20% of the first blood pressure determined.

At arrival in the recovery room patients will be monitored by pulse oximetry (SO_2_), ECG and NIBP only.

The level of recovery from anesthesia and the return of psychomotor fitness will be assessed using the modified Aldrete Score directly at the end of the procedure, at arrival in the recovery room, 30 and 60 min later. All patients will stay in the recovery room for at least two hours. Ready for virtual discharge will be declared when an Aldrete Score ≥ 9 or pre-procedure score is met.

“Discharge criteria” require further that the patient is awake and alert with stable vital signs, is able to ambulate without assistance, and is free of side effects of the drugs employed during the procedure.

### Pain evaluation

To classify the pain score the Society of Critical Care Medicine recommends self-reporting by conscious sedated patients using the numerical rating scale (NRS, range 0–10) [11]. So we decided to use the NRS to estimate patients’ pain. However, we had to recognize, that the use of a self reporting score -even in only conscious sedated patients- is sometimes complicated and unreliable because of lack of comprehension and communication tools. Therefore, we decided to use a modified version of the Behavioural Pain Scale for not-intubated patients (BPS-NI) [12] as an additional rating method to assess the pain level among patients with altered level of consciousness. The BPS – IN evaluates three behavioural domains (i.e., facial expression, movements of upper limbs and vocalization). Each domain contains four descriptors which are rated on a 1–4 scale, and the total BPS value ranges from 3 (no pain) to 12 (most pain).

Patients with a NRS > 4 or BPS-NI ≥ 7 will be given an dose of 100 μg alfentanil.

The amount of alfentanil and propofol used as an adjunct for conscious sedation will also be recorded as part of this study.

### Measurements

#### ***Trieger test***

Patients have to accomplish the Trieger test (before and 30 minutes after the procedure) as a means of psychomotoric recovery [13]. For the Trieger test, patients have to connect points forming a figure with a line using a pen. Missing points as well as the distances of the line from the true points are noted and rated as score post/preprocedural.

### Questionnaires

Before procedure patients and GE’s have to fill in a questionnaire concerning basic information and specific expectations of the procedure.

Following the procedure and before discharge, patients and gastroenterologist are urged to fill in questionnaires modified from the Patient Satisfaction with Sedation Instrument (PSSI), respectively Clinical Satisfaction with Sedation Instrument (CSSI), which provide feasible, reliable, and valid assessment of procedural sedation satisfaction for outpatients [14]. Vargo et al. [14] developed this score system with finally 4 subscores to describe patients and 3 subscores to describe endoscopists’ satisfaction. Subscores for the PSSI contain questions about sedation delivery, procedural recall, sedation side effects, and global satisfaction. Subscores for the CSSI refer to corresponding issues among gastroenterologists: Sedation administration, recovery/ post-op and global satisfaction.

Patients and endoscopists could classify their satisfaction or dissatisfaction during the procedure on a questionnaire ranging from 1 = very satisfied to 7 = very dissatisfied.

A follow-up telephone call will be made 24 h later asking questions from part two of the PSSI questionnaire concerning global satisfaction. Patients will also be asked about their willingness to undergo the same procedure with the same sedation regimen in the future if required.

### Objectives

#### ***Primary objective***

The primary aim of this prospective randomized controlled study is to determine, whether dexmedetomidine is more effective for sedation during endoscopic oesophageal interventions than our in house standard regime with propofol TCI. Surrogate marker of effectiveness are satisfaction levels, pain score, and sedation score measured by questionnaires which have to be completed by both, patient and GE, and patients sweat production as a sign of stress response.

#### ***Secondary objective***

As a secondary objective we set out to clarify, whether dexmedetomidine sedation is safer for the patient with regard to respiratory and cardiovascular side effects than sedation with propofol TCI. Surrogate parameters of pulmonary and cardiovascular events are a decline in oxygen saturation (SO_2_) to less than 90%, a rise in exCO_2_ > 50 mmHg, or a respiratory rate < 6/min; heart rate less or more than 20% of the baseline rate or occurrence of an arrhythmia; blood pressure less or more than 20% of the first pressure measured.

### Sample size

Regarding the primary outcome parameter effectiveness and satisfaction, our trial is set up as a study of a continuous response variable from two independent subjects. Sample size calculation is based on a former study (PAGE- study, ISRCTN 83950185). This study shows significant and thus important differences between the study-groups within the validated patient and gastroenterologists questionnaires. Within these questionnaires the maximum number of points patients or endoscopists can award, is seven reaching from 1 (highly satisfied) to 7 (very dissatisfied). With a true difference between two groups of 0.5 a sample size of 27 subjects per group was necessary to demonstrate a statistical significance for global satisfaction (patient and gastroenterologist) at an α of 0.05 and a 1-β of 0.8. If the calculated drop out is 20%, the estimated sample size will be 32 patients per group.

### Statistical analysis

Statistical analyses will be performed using PASW statistics (version 18.0).

All data will be checked for normal distribution using the Kolmogorov-Smirnonov test. The two groups will be compared using a Student *t* test or multifactorial ANOVA (Kruska Wallis) with a step-down post hoc adjustment, or a Mann–Whitney *U*-test (for differences between two groups), where appropriate. Data are presented as mean ± SD or median/25/75 percentile.

### Ethical approval

Ethical approval was obtained from the Medical Ethics Committee of the Academic Medical Center, Amsterdam, the Netherland (NL). A marginal review was performed by the National Authority, the Central Committee on Research Involving Human Subjects (CCMO), and there were no objections to perform this study (NL36861.018.11).

## Discussion

Mapping, ER, and RFA are nowadays widespread used as a treatment for early neoplastic oesophageal or gastric lesions. However, it is still a matter of discussion which form of sedation is necessary and safe: conscious or deep sedation.

Propofol TCI combined is a tool for deep sedation, with high levels of acceptance and satisfaction among patients and gastroenterologist, but at a great risk of cardiopulmonary depression. This is the reason why its application in many countries is still limited to anesthesia providers. Dexmedetomidine is a highly selective alpha_2_ agonist that provides anxiolyse and cooperative sedation without respiratory depression. Hashiguchi [15] performed a study (n = 40) to investigate the safety and efficacy of dexmedetomidine for sedation of patients undergoing routine upper gastrointestinal endoscopy. The results of this randomized study demonstrate that dexmedetomidine was as safe and effective as midazolam for producing and maintaining adequate short-term sedation in patients undergoing upper GI endoscopy. Unfortunately, length and sort of upper GI were not described.

Takimoto [16] compared dexmedetomidine with propofol and midazolam for sedation of 90 patients during endoscopic submucosal dissection of gastric cancer and found dexmedetomidine safe and effective. However, in this study only HR, NIBD, ECG and SO2 were monitored in intervals of 10 min and patient and endoscopist satisfaction was not mentioned.

Mazanikov et al. [17] used dexmedetomidine for sedation of patients with chronic alcohol abuse during ERCP and found an insufficient sedative effect.

This RCT is the first one with a broad hemodynamic monitoring including exCO_2_, NICO and sweat conductance/stress monitoring and validated questionnaires on patient and endoscopists satisfaction. So, this study will provide detailed information about the effect of dexmedetomidine concerning procedural sedation aspects, side effects, adverse events, procedural time and recovery time.

We expect that dexmedetomidine is a safe and effective possibility to run through oesophageal endoscopic procedure with satisfied patients and endoscopists.

## Abbreviations

BPS-NI: Behavioral pain scale for not intubated patients; CSSI: Clinical satisfaction with sedation instrument; EMR: Endoscopic mucosal resection; ExCO2: Exhaled CO2; HR: Heart rate; NIBP: Non-invasive blood pressure; NICO: Non- invasive cardiac output; NRS: Numeric rating score; OAA/S: Observer’s assessment of alertness/sedation; RFA: Radiofrequency ablation; PSSI: Patient satisfaction with sedation instrument; SO2: Oxygen saturation; TCI: Target controlled infusion.

## Competing interests

The authors declare that they have no competing interests.

## Authors’ contributions

SE is responsible for drafting the manuscript. SE, MWH, and BP are responsible for the study design. SE, MWH, BP, and JJB are responsible for revising the manuscript. All authors have read and approved the manuscript.

## Pre-publication history

The pre-publication history for this paper can be accessed here:

http://www.biomedcentral.com/1471-230X/13/176/prepub
